# Dispensary Abuses. Addressed to the Profession

**Published:** 1834-10-01

**Authors:** 


*Dispensary Abuses. Addressed to the Profession.* London, 1834. Pp. 8.

Our readers will observe that, in quoting the title of the important work before us, we have deviated from our usual custom, and have omitted to mention its size. Now this omission depends on a most cogent reason: there are no signatures to the book, and we have not a folder's eye, to determine at a glance whether it be in thirty-twos or sixty-fours. We may assist the student, however, in forming a notion of this unnamed size, by observing that it tallies with that of Bob Short's Rules for Whist. Not that we would have people suppose that there is any other resemblance between the two books: far from it; the one is a calm scientific treatise, the other an unsparing philippic. The sober Robert indeed does once sarcastically desire us to keep our temper, as if our temper was likely to follow our silver threepences; but the great unknown before us is Nemesis in the shape of a pamphlet in sixty-fours.

We shall now proceed to pirate all, or the greater part of the admonitions before us; and shall commence with an extract amounting to about three-eighths of the whole.

"Sir: London Dispensaries, as at present constituted, are an absolute nuisance, and peculiarly injurious to the poor, as well as the just claims and respectability of medical men of education and experience. Indeed, those members of the profession who shall in future persist in attending such establishments, should be visited with the marked displeasure of the profession at large. From the commencement of your career, up to the present moment, your arduous and extraordinary exertions, which involve the health of British subjects in every corner of the world, have produced the most beneficial effects in many ways to the cause of humanity. But these admonitions, so fervently and clearly demonstrating the incalculable mischiefs arising out of such institutions to medical men and their families, have been so shamefully and ungratefully neglected, that we are inclined to believe that a species of fatuity exists among professional men, which ought to be called a medical dispensary monomania, and will authorise you, on application, to sign their certificates as just objects for admission into certain establishments, should they continue to adhere to their present course. Your efforts, so laudable in the eyes of reflecting men, have been heretofore under this department of flagrant abuse so much neglected, that we have been compelled to adopt the opinion, that these valuable exertions resolve themselves as it were into the throwing of pearls before the abhorred of the Jews. A meeting therefore of the profession on the subject of the attendance on dispensaries, which must be regarded as a species of medical *felo de se*, would be of admirable utility at the present juncture; as it would give vigour to the virtuous members of the profession, and bring the delinquents and evils arising from the vile dispensary system, which originated in that nest of fraud, the Colleges of Physicians and Surgeons, under the eye of the profession, and the nation at large. The receiving-rooms of dispensaries, into which diseases of a highly contagious nature are daily admitted, become the sources of direful evil to the community, because from thence as from a centre infectious maladies of a most inveterate character are diffused over every part of the nation where they exist, and thus they become sources of great expense, as well as of tragical events, as domestics convey these fatal diseases to the highest circles. The history of dispensaries and their abuses, by which medical men suffer most severely, would excite the astonishment and indignation of every virtuous and generous mind, by unfolding their defects. These institutions, in defiance of all your persuasive exertions, lamentable to be said, still continue to be anxiously sought after by medical men, greatly to their own degradation." (P. 1.)

The word *Sir* at the beginning might induce some of your quick people, who recklessly jump at a conclusion, without thought or inquiry, to suppose that this letter is a circular: no such thing. We learn from the P.S. that "the above was addressed to the Editor of a distinguished Journal, and hence its appearance in the original form." For our parts, we cannot recollect the appearance of this letter in any of the distinguished Journals with which this island is blessed, and we therefore conjecture that it must have been published in some 64mo. Journal, known only to a select few.

It is melancholy to reflect how difficult it is to reform the world. Here, for instance, the editor of the unknown distinguished Journal has been wasting the midnight oil, and scores of patent pens, in endeavouring to teach doctors how to grow rich; but they have all refused to learn, to a man. Dispensaries and small beer for ever! has been their cry, when the editor was willing to give them claret and private practice. We do not wonder then that our pamphleteer proposes that the editor should send the afflicted dispensarians to "certain establishments." The act of parliament indeed requires *two* signatures, but mere formal difficulties of this kind are soon got over. Besides, a distinguished Journal probably has a sub-editor, and a number of blank certificates, with the two signatures ready written, might be kept waiting in some snug pigeon-hole, for the benefit of any doctors who should exhibit symptoms of a more than usual dispensarian friskiness. We admire our author's candour, in telling his friend that his exertions "resolve themselves, as it were, into the throwing of pearls before the abhorred of the Jews." We do not think his friend will like it, though. Nobody loves to be reminded that he has been engaged in washing blackamoors white, or twisting ropes of sand, or making silk purses out of the ears of "the abhorred of the Jews," or transmuting dispensary doctors into "virtuous members of the profession." The meeting, however, that our author talks of should be convened without the loss of a day; for it would not only be the parent of what would be called in America an anti-dispensary excitement, but might even be the forerunner of a general professional turn-out. The chair, we think, ought to be taken by a retired dispensary physician, on the principle that your reformed sinner makes the finest saint. We recollect that, some five or six years since, an excellent newspaper congratulated its readers that the cabinet was purged of the last taint of liberality: now, with the aid of the medical meeting in question, we think that the same foul canker might be cut out of our profession. We use these strong expressions advisedly; for, if the dispensary leeches will persist in their ruinous liberality, and shameful gratuitous advice, measures must be taken accordingly. If they will not listen to the friendly admonitions of the unknown Journal, they must be put to open shame.

> "Cuncta prius tentanda, sed immedicabile vulnus
> Ense recidendum est, ne pars sincera trahatur."

Our author complains (and with great justice) of the dispensary doctors, for diminishing the general funds of the profession, by seeing the poor gratis; for it would be foolish to say that they have no money to give in fees. Could they not give money's worth in clothes, victuals, and small articles of furniture? Nevertheless, it is certain that our author has furnished the delinquents with a defence which they will not fail to make use of. They will assert, (and will quote his pamphlet as irrefragable authority,) that what the profession loses directly, it more than gains indirectly, by the dispensary receiving-rooms becoming centres of infection, and, in consequence, "sources of great expense, as well as of tragical events." If the profession loses five guineas by Mrs. Finikin's gratuitous cure, doth it not gain a hundred by the infection being carried to my Lady?

We must content ourselves with one more quotation.

"Does the lawyer or the clergyman give assistance gratuitously to either poor or rich? Does the grocer, the baker, the brewer, the butcher, apply his goods to the support of those institutions? Certainly not; because they are well aware that, if *they* were to do so, they would be regarded as maniacs, or considered disgracefully inattentive to their own interests and that of their families. And were such goods offered, it would be difficult to persuade even the poor to make use of them; not only because of the disgrace of doing so, as taking advantage of an inconsiderate fool, but from the conception that the articles so distributed were unsound and worthless." (P. 5.)

It certainly is strange that no infatuated tradesmen have ever supplied their goods gratis. They would be very acceptable. The grocer might send his figs, (to make poultices,) the butcher his steel, the baker his alum, and the brewer his quassia. It is with great diffidence that we differ from a man who shows so profound a knowledge of the world as our author, yet we think that the poor *might* be coaxed into receiving other things gratis, as well as physic. We would bet him a handful of fees, that, if he will set up a gratuitous butcher's shop, in a quiet way, cutting up an ox or so per day, he will be able to do a good business, and will dispose not only of his edgebones and sirloins, but even of his clods and sticking-pieces.

We must now take leave of our author, giving him our hearty thanks for the composition of this letter. It is refreshing to know that there is such a man in the world.

